# Routine HIV Testing in Botswana: A Population-Based Study on Attitudes, Practices, and Human Rights Concerns

**DOI:** 10.1371/journal.pmed.0030261

**Published:** 2006-07-18

**Authors:** Sheri D Weiser, Michele Heisler, Karen Leiter, Fiona Percy-de Korte, Sheila Tlou, Sonya DeMonner, Nthabiseng Phaladze, David R Bangsberg, Vincent Iacopino

**Affiliations:** **1**Physicians for Human Rights, Cambridge, Massachusetts, United States of America; **2**Center for AIDS Prevention Studies, University of California San Francisco, San Francisco, California, United States of America; **3**Epidemiology and Prevention Interventions (EPI) Center, Division of Infectious Diseases, San Francisco General Hospital, University of California San Francisco, San Francisco, California, United States of America; **4**Veterans Affairs Ann Arbor Health System and Department of Internal Medicine, University of Michigan School of Medicine, Ann Arbor, Michigan, United States of America; **5**Department of Nursing, University of Botswana, Gaborone, Botswana; **6**Positive Health Program, San Francisco General Hospital, University of California San Francisco, San Francisco, California, United States of America; **7**Department of Medicine, University of Minnesota, Minneapolis, Minnesota, United States of America; University of AmsterdamNetherlands

## Abstract

**Background:**

The Botswana government recently implemented a policy of routine or “opt-out” HIV testing in response to the high prevalence of HIV infection, estimated at 37% of adults.

**Methods and Findings:**

We conducted a cross-sectional, population-based study of 1,268 adults from five districts in Botswana to assess knowledge of and attitudes toward routine testing, correlates of HIV testing, and barriers and facilitators to testing, 11 months after the introduction of this policy. Most participants (81%) reported being extremely or very much in favor of routine testing. The majority believed that this policy would decrease barriers to testing (89%), HIV-related stigma (60%), and violence toward women (55%), and would increase access to antiretroviral treatment (93%). At the same time, 43% of participants believed that routine testing would lead people to avoid going to the doctor for fear of testing, and 14% believed that this policy could increase gender-based violence related to testing. The prevalence of self-reported HIV testing was 48%. Adjusted correlates of testing included female gender (AOR = 1.5, 95% CI = 1.1–1.9), higher education (AOR = 2.0, 95% CI = 1.5–2.7), more frequent healthcare visits (AOR = 1.9, 95% CI = 1.3–2.7), perceived access to HIV testing (AOR = 1.6, 95% CI = 1.1–2.5), and inconsistent condom use (AOR = 1.6, 95% CI = 1.2–2.1). Individuals with stigmatizing attitudes toward people living with HIV and AIDS were less likely to have been tested for HIV/AIDS (AOR = 0.7, 95% CI = 0.5–0.9) or to have heard of routine testing (AOR = 0.59, 95% CI = 0.45–0.76). While experiences with voluntary and routine testing overall were positive, 68% felt that they could not refuse the HIV test. Key barriers to testing included fear of learning one's status (49%), lack of perceived HIV risk (43%), and fear of having to change sexual practices with a positive HIV test (33%).

**Conclusions:**

Routine testing appears to be widely supported and may reduce barriers to testing in Botswana. As routine testing is adopted elsewhere, measures should be implemented to assure true informed consent and human rights safeguards, including protection from HIV-related discrimination and protection of women against partner violence related to testing.

## Editors' Summary

**Background.:**

In 2005, there were 5 million new infections with the human immunodeficiency virus (HIV), and the disease it causes—acquired immunodeficiency syndrome (AIDS)—killed three million people. Despite the increased availability of drugs that can fight HIV (antiretrovirals), the AIDS epidemic continues to grow, particularly in sub-Saharan Africa. To halt it, more needs to be done to prevent the spread of HIV. Education about safe sex can help—HIV is most commonly spread through unprotected sex with an infected partner—but increasing HIV testing is of paramount importance. Unfortunately, the uptake of voluntary counseling and testing in sub-Saharan Africa is worryingly low. Fear of being stigmatized—socially disgraced—and discriminated against, fears about the positive result itself, and worries about access to antiretroviral drugs are all putting people off being tested.

**Why Was This Study Done?:**

In Botswana, one in three adults is infected with HIV. Since 2002, antiretroviral drugs have been freely available but enrollment in the Botswana National Treatment Program during its first two years was slow, in part due to inadequate uptake of voluntary HIV testing. Consequently, in early 2004, the government introduced a policy of routine HIV testing in which all patients are tested for HIV when they visit their doctor unless they opt out. A major aim of this approach to HIV testing, which was formally recommended in June 2004 by UNAIDS and the World Health Organization, is to increase uptake of HIV testing and treatment, and to reduce HIV-related stigma by treating the HIV test like any other routine medical procedure. However, there are fears that the policy could back-fire—people might not visit their doctors, for example, because they are afraid of being tested and think that they will not be able to refuse the test. In this study, the researchers investigated knowledge of and attitudes to routine testing in Botswana to understand better the consequences of a routine testing policy.

**What Did the Researchers Do and Find?:**

The researchers interviewed adults throughout Botswana about their knowledge of and attitudes to routine HIV testing 11 months after introduction of the policy. Only half of the participants had heard of routine testing before being interviewed but nearly all were in favor of routine testing. More than half thought it would reduce HIV-related stigma and the violence toward women that is associated with an HIV-positive status. However, almost half believed that routine testing might prevent people from going to the doctor because of fear of testing and a few thought the policy would increase violence against women. Nearly half of the interviewees had had an HIV test and the researchers found, for example, that women were more likely to have been tested than men and that people with stigmatizing attitudes toward people living with HIV and AIDS were less likely to be tested. Fear of learning one's HIV status, lack of perceived risk, and fear of having to change sexual practices if positive all stopped people taking the test. Finally, although experiences with testing were generally positive, approximately two-thirds of interviewees who had been tested felt that it would have been difficult to refuse the test.

**What Do These Findings Mean?:**

These results show that there is widespread support for routine HIV testing in Botswana, a finding supported by recent increases in treatment uptake. Routine testing, write the researchers, holds significant promise for the prevention and treatment of HIV/AIDS in Botswana and elsewhere. In particular, increasing the number of people tested for HIV may reduce HIV-related stigma, which should further increase testing and hopefully slow the spread of HIV. But the results of this study also highlight some areas of concern. Whenever HIV testing policies are implemented, human rights must be protected by ensuring that patients have all the information necessary to make an informed and free decision about being tested, by providing protection for women against violence related to HIV status, and by ensuring total confidentiality. Careful monitoring of Botswana's program and similar programs will be needed to ensure that these human rights are fully met, conclude the researchers.

**Additional Information.:**

Please access these Web sites via the online version of this summary at http://dx.doi.org/10.1371/journal.pmed.0030261

• US National Institute of Allergy and Infectious Diseases factsheet on HIV infection and AIDS

• US Department of Health and Human Services information on AIDS

• US Centers for Disease Control and Prevention information on HIV/AIDS

• UNAIDS and World Health Organization 2004 policy statement on HIV testing

• AVERT, a UK-based charity, provides information about HIV and AIDS in Botswana

## Abstract 3

A cross-sectional, population-based study of 1,268 adults from five districts in Botswana showed that routine HIV testing appears to be widely supported and may reduce barriers to HIV testing.

## Introduction

There has been widespread concern about the slow uptake of voluntary counseling and testing (VCT) in many parts of sub-Saharan Africa [ 1, 2]. VCT is a cornerstone of cost-effective HIV prevention and linkage to expanding HIV treatment in low-resource settings [ 3, 4]. Some of the most significant barriers to HIV testing identified in sub-Saharan Africa include lack of access to VCT and to high quality clinical services, lack of access to antiretroviral (ARV) therapy, and HIV-related stigma [ 1, 5, 6].

With a seroprevalence of 37% of adults ages 15–49 [ 7, 8], Botswana established universal access to antiretroviral treatment (ART) beginning in 2002 for all patients with CD4 counts less than 200 or with an AIDS-defining illness [ 9– 11]. By January 2004, however, only 17,500 patients were enrolled in the Botswana National Treatment Program out of an estimated 110,000 eligible individuals [ 9]. Slow enrollment in HIV treatment was thought to be due in part to underutilization of HIV testing [ 9, 11, 12]; by mid-2003, only 70,000 tests in total had been performed in Botswana out of a population of 1.7 million [ 13]. HIV stigma was identified by government and press sources as one possible impediment to HIV testing and hence to the success of the new ART program, in that individuals may avoid testing and treatment facilities to avoid potential stigma and discrimination [ 8, 11, 13]. We previously reported that social stigma and fear of positive test results significantly delayed testing among a group of patients treated in the private sector in 2000 [ 14].

In an attempt to increase the uptake of HIV testing and ART, the Botswana government introduced the policy of routine HIV testing in early 2004, whereby nearly all patients would be tested as a routine part of medical visits unless they explicitly refused [ 13, 15]. While this approach to testing is provider-initiated, all patients should receive essential information about HIV testing and be informed of their right to refuse. In addition, there is typically greater emphasis on post-test compared with pre-test counseling [ 16]. Studies in resource-rich settings have shown that routine HIV testing can be cost-effective and life-saving, both by increasing the life expectancy of individuals with HIV and by reducing the annual HIV transmission rate [ 17– 21]. In June 2004, as part of a change in testing policy recommendations, UNAIDS and the World Health Organization recommended the routine offer of HIV testing by healthcare providers in a wide range of clinical encounters based in part on the Botswana experience [ 22, 23]. The goal of routine testing is to increase the proportion of individuals aware of their status, and thereby reduce “HIV exceptionalism,” lessen HIV-related stigma, and provide more people access to life-saving therapy [ 16, 24]. While provider-initiated approaches to testing are gaining popularity, there have been concerns that routine testing policies are potentially coercive, that counseling will no longer be practiced, that people may be dissuaded from visiting their doctors for fear of being tested, and that this policy may increase testing-related partner violence [ 15, 25– 27].

As routine testing is increasingly being recommended as an option in other countries [ 17, 18, 28– 30], it is important to improve our understanding of the consequences and specific human rights concerns associated with implementation of this policy in Botswana. We therefore assessed: 1) knowledge of and attitudes toward routine testing in Botswana with a focus on human rights concerns associated with this policy; 2) factors associated with whether respondents had heard of routine testing, and with positive attitudes toward this policy; and 3) the prevalence and correlates of HIV testing, barriers and facilitators to testing, and reported experiences with testing 11 months after the introduction of routine testing in Botswana.

## Methods

In November and December 2004, we conducted a cross-sectional study using structured survey instruments among a probability sample of 1,268 adults selected from the five districts of Botswana with the highest number of HIV-infected individuals. These districts included Gaborone, Kweneng East, Francistown, Serowe/Palapye, and Tutume, and cover a population of 725,000 out of a total population of 1.7 million individuals in Botswana. We used a stratified two-stage probability sample design for the selection of the population-based sample with the assistance of the Central Statistics Office at the Ministry of Finance and Development Planning in Botswana. In the first stage of sampling, 89 enumeration areas were selected with probability proportional to measures of size, where measures of size are the number of households in the enumeration area as defined by the 2001 Population and Housing Census. At the second stage of sampling, households were systematically selected in each enumeration area by trained field researchers under the guidance of field supervisors. With a target sample of 1,200 households, and 15% over-sampling for an anticipated 85% response rate, 1,433 households were selected. Within each household, random number tables were used to select one adult member who met our inclusion criteria, and up to two repeat visits were made.

Participants were excluded if they were older than 49 or younger than 18 years old, if they had cognitive disabilities, or if there was inadequate privacy to conduct the interviews. The 45- to 60-minute survey was conducted in either English or Setswana in a private setting, and written consent was obtained from all study participants. Our structured survey instrument and consent forms were pilot-tested among 20 individuals from Gaborone, and then translated into Setswana and back-translated into English. All study procedures were approved by the Human Subjects Committee at the University of California San Francisco (San Francisco, California, United States), as well as by the Botswana Ministry of Health Research and Development Committee.

### Measures

**Figure 1:**
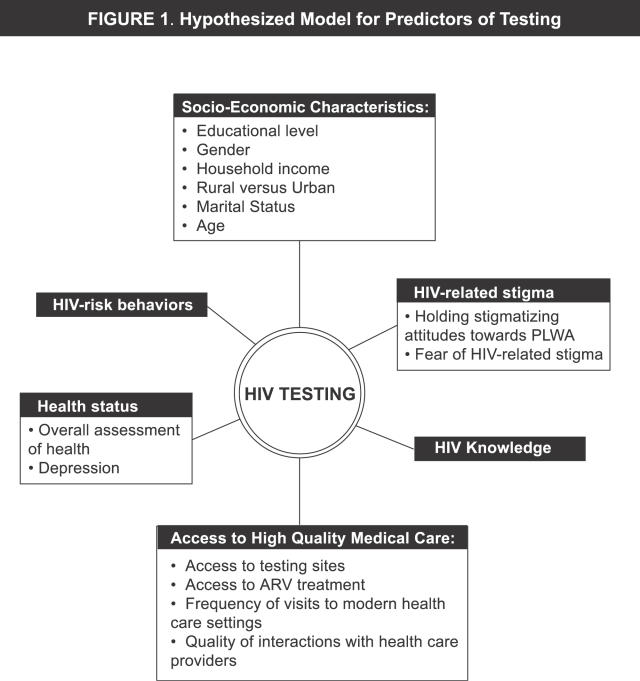
Hypothesized Model for Predictors of Testing

Domains of inquiry for our 234-item survey ( Protocol S1) included demographics, HIV knowledge, experiences with HIV testing, barriers and facilitators to HIV testing, attitudes toward routine testing, HIV risk behaviors, HIV-related stigma, depression, beliefs about gender roles and gender discrimination, and measures of healthcare access and utilization. Based on an extensive literature review [ 2, 6, 31– 37] and discussions with key informants, we developed a conceptual model that guided the selection of variables for our multivariate model for correlates of testing, as shown in Figure 1. Relevant variables are explained below.

#### Knowledge of and attitudes toward routine testing.

Participants were asked whether they had heard of routine testing and were given a detailed explanation of this policy (see Table 1). Participants then indicated the extent to which they are in favor of routine testing and whether they think this policy affects HIV-related stigma, barriers to testing, violence against women related to testing, and uptake of ARVs. From questions assessing attitudes toward routine testing ( Table 1), we constructed an ordinal outcome of positive attitudes toward this policy. Participants were categorized as having zero to one, two, three, or four positive views toward routine testing. (See Tables 1 and 2 for specific items.)

**Table 1:**
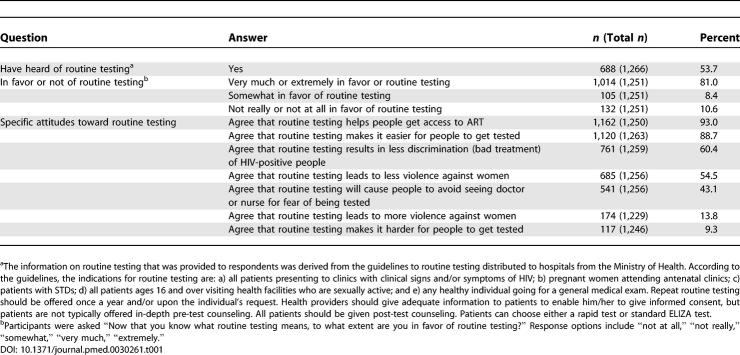
Attitudes toward Routine Testing

**Table 2:**
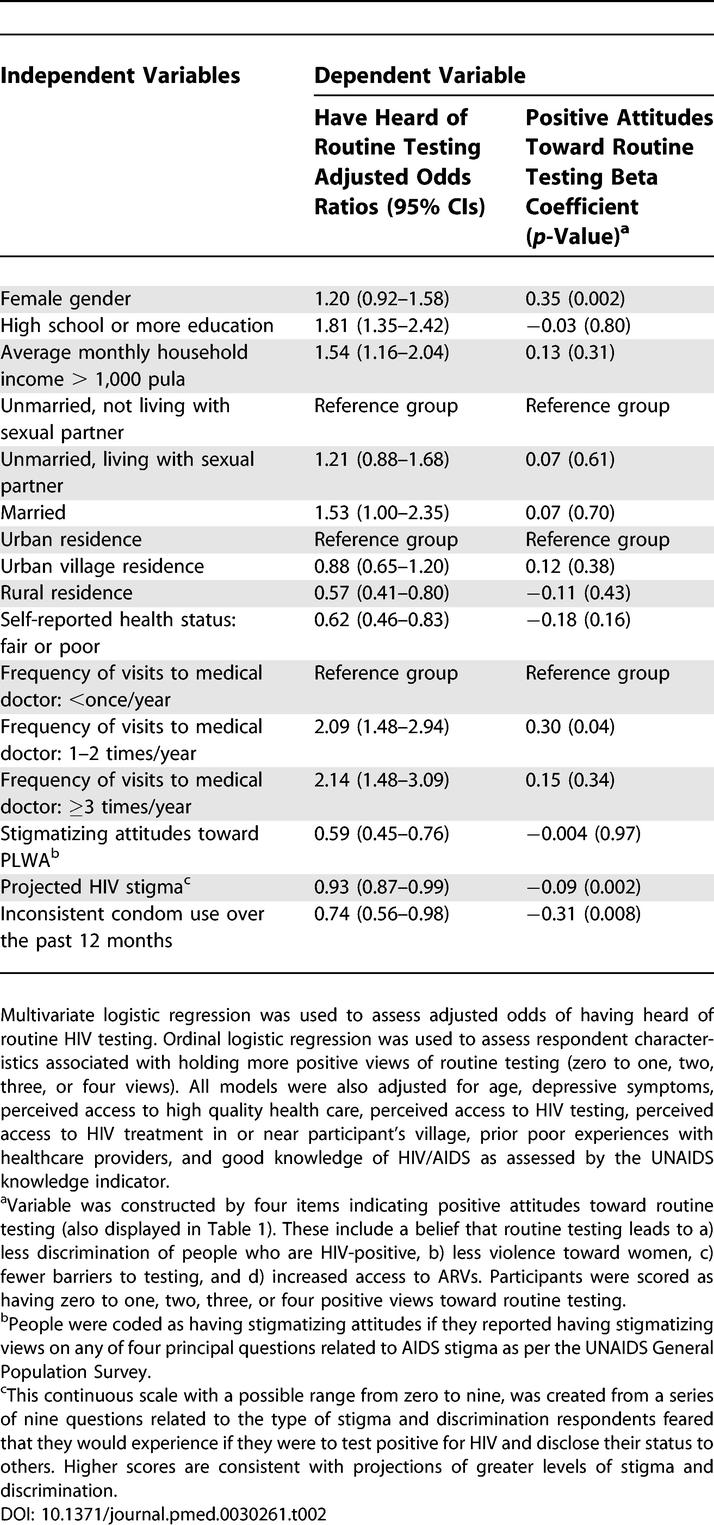
Respondent Characteristics Associated with Having Heard of Routine Testing and with Positive Attitudes Toward Routine Testing in Multivariate Analyses ( *n* = 1,168)

#### HIV testing.

Participants were asked whether they had ever been tested for HIV (by either VCT or routine testing). If so, they were asked detailed questions about their experiences with pre-test and post-test counseling, confidentiality, facilitators to testing, and personal repercussions of testing. If not, they were asked a series of questions related to barriers to testing adapted from the CDC HIV Testing Instrument, version 9.00, and about their intention to be tested within the next six months. HIV status was not asked in order to maximize response rate and hence the generalizability of the population-based sample.

#### HIV-related stigma.

Respondents were asked seven questions representing potential stigmatizing attitudes adapted from the UNAIDS general population survey and the Department of Health Services AIDS module, which have been used successfully in previous studies in Botswana [ 38]. Anyone who reported a discriminatory attitude on any of four principal questions was registered as having stigmatizing attitudes per the UNAIDS scoring system. Since participants may not always openly endorse stigmatizing views toward people living with HIV and AIDS (PLWA) due to social desirability bias, as an additional measure of stigma, we also asked individuals to project the type of responses they would anticipate from others if they were to test positive for HIV and divulge their status to others. We converted this information to a nine-item index on “projected HIV stigma” with higher scores associated with a greater number of reported adverse social consequences associated with testing positive. This index had high internal reliability with a Cronbach alpha of 0.77.

#### HIV knowledge.

Participants were asked 15 questions about their knowledge of HIV transmission and prevention, based on questions modified from the UNAIDS General Population Survey and the Department of Health Services AIDS module. Using the UNAIDS knowledge indicator scoring system, individuals were scored as having HIV knowledge if they correctly identified the two most common modes of HIV prevention in Botswana.

#### Depression.

As depression is known to impede access to care and to worsen HIV outcomes in Western settings, we included depression in our analysis [ 39– 41]. Symptoms of depression were measured using the 15-item Hopkins Symptom Checklist for Depression [ 42] which has been validated previously in locations in Africa and elsewhere [ 43].

### Analysis

We used standard procedures for data entry and quality control. All data were analyzed with S TATA statistical software. Outcomes of interest included: a) having heard of routine testing; b) number of positive attitudes toward routine testing (categorized as an ordinal variable consisting of the following categories: zero to one, two, three, and four positive statements about routine testing); c) self-reported HIV testing (by either VCT or routine testing); d) having been tested under routine testing; and e) planning to test within the next six months (among people who had not tested).

The following covariates were included in our analyses: 1) age (continuous); 2) sex; 3) income (≥population mean, <population mean); 4) education (≥high school, <high school); 5) residence type (rural, urban, urban village); 6) marital status (married, living with partner, other); 7) knowledge surrounding HIV/AIDS; 8) HIV-related stigma; 9) symptoms of depression (dichotomous variable); 10) frequency of visits to a medical provider (<once/year, 1–2 times/year, ≥3 times/year); 11) perceived access to good quality medical clinics; 12) access to ARV therapy in the respondent's community; 13) access to HIV testing sites; 14) projected HIV stigma (continuous index); 15) history of not being consistently treated with respect by health providers; 16) health status (very good or good versus fair or poor); and 17) history of inconsistent condom use over the past year.

We used descriptive statistics to examine sample characteristics and experiences with and attitudes toward testing. We then conducted univariate and multivariate logistic regression analyses to explore the independent association of each covariate with having heard of routine testing, having had a prior HIV test, having been tested for HIV by routine testing, and planning to test for HIV among those not tested. We used ordinal logistic regression to assess factors associated with number of positive attitudes toward routine testing. All variables from our conceptual model were included in our final multivariate models. Regression diagnostic procedures yielded no evidence of multi-collinearity or overly influential outliers in any of the models. No variable had more than 3% missing data.

## Results

### Description of Study Population

1,268 (89%) respondents completed the survey. Among 165 non-respondents, 46 (28%) were unavailable after two repeat visits, 78 (47%) refused or did not meet criteria, and 41 (25%) were unable to complete the interview. Demographic and behavioral characteristics of the study population are shown in Table 3.

**Table 3:**
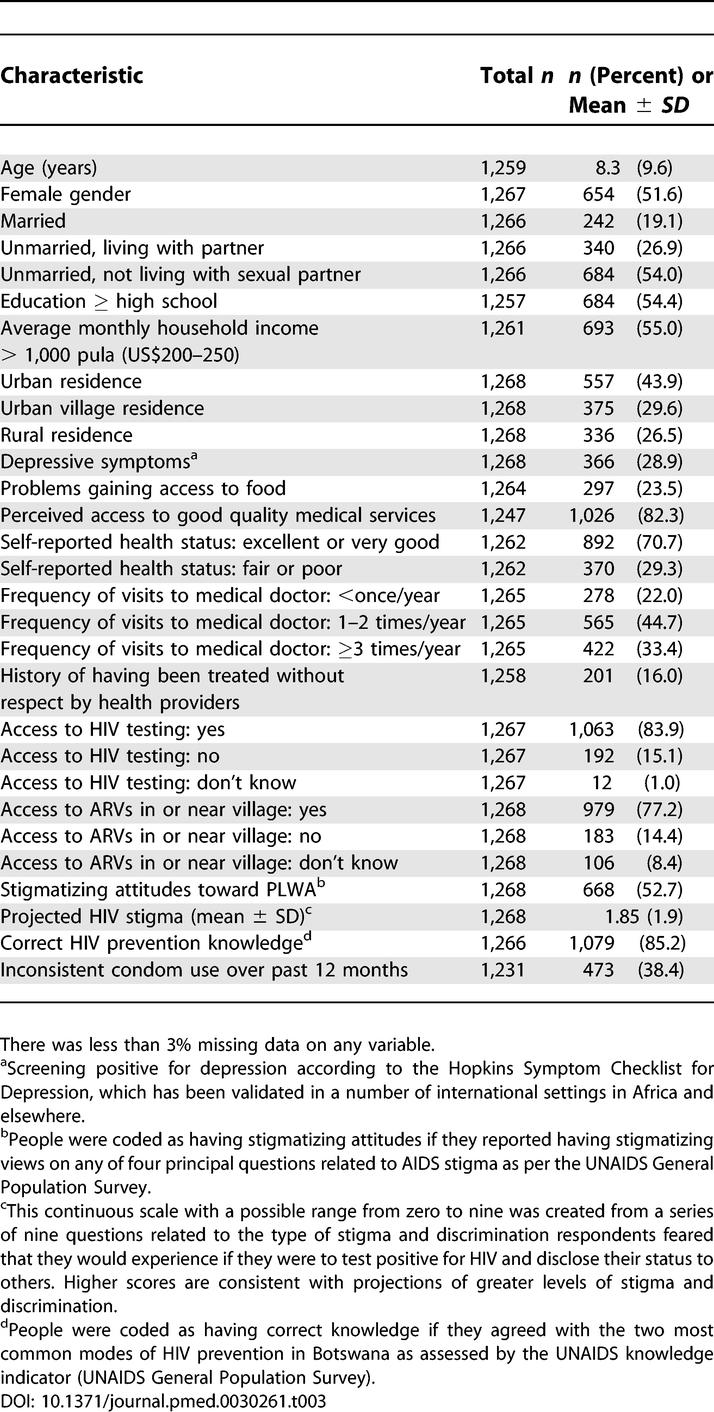
Baseline Characteristics of Respondents ( *n* = 1,268)

### Knowledge of and Attitudes toward Routine Testing

Fifty-four percent of respondents had heard of routine testing before the survey interview ( Table 1). After adjusting for all independent variables (see Figure 1), higher education, higher income, being married, having better health, and having more frequent medical visits were each associated with higher odds of having heard of routine testing ( Table 2, column 2). Respondents who reported stigmatizing attitudes toward PLWA had lower odds of having heard of routine testing (AOR = 0.59, 95% CI = 0.45–0.76), as did respondents with more fears of being stigmatized if they tested positive, people in rural areas, and people who reported inconsistent condom use.

### Attitudes toward Routine Testing

Although approximately half of respondents had heard of routine testing before the interview, a majority endorsed positive views toward routine testing after the policy was explained ( Table 1). Eighty-one percent were “very much” or “extremely” in favor of routine testing. A majority agreed that routine testing results in decreased discrimination of HIV-positive people (60%), leads to decreased violence against women (55%), and makes it easier for people to get tested (89%) and to gain access to ART (93%). On the other hand, 43% believed that routine testing would cause people to avoid seeing their health provider for fear of being tested, and 14% thought that routine testing would lead to more violence against women. There were fewer than 2% mutually incompatible response pairs in each of our questions on routine testing.

In ordinal logistic regression analyses, with number of positive views toward routine testing as the outcome variable ( Table 2, column 3), those with more fears of being stigmatized themselves if they tested positive for HIV had significantly fewer positive views than those without such fears. Similarly, those who reported unsafe sexual practices had fewer positive attitudes.

### Prevalence and Correlates of Having Been Tested for HIV

Overall, 605 respondents (48%) reported that they had been tested for HIV. The proportion tested according to demographic and behavioral attributes are shown in Table 4. Factors associated with having been tested in unadjusted analyses included: older age, female gender, higher education, higher income, self-reported excellent or good health status, more frequent medical visits, access to good healthcare, access to HIV testing, being treated with respect consistently by health providers, lack of stigmatizing attitudes toward PLWA, and consistent condom use ( Table 4). In adjusted analyses, associations remained among all these covariates and HIV testing except for older age, higher income, and being treated with respect consistently by health providers.

**Table 4:**
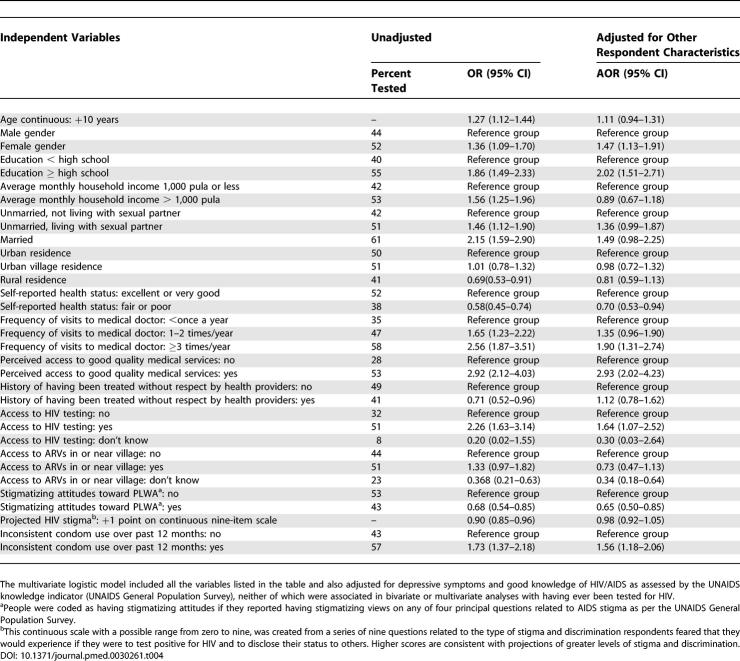
Unadjusted and Adjusted Odds of Having Been Tested for HIV ( *n* = 1,164)

Fifteen percent of tested respondents reported having been tested by routine testing. In a multivariate logistic regression model with being tested by routine testing as the dependent outcome, and including all covariates listed in Figure 1, the only independent correlates of getting routine testing were being married (AOR = 2.67, CI = 1.29–5.53) and seeing the medical provider more than three times per year (AOR = 2.95, CI = 1.41–6.20). In addition, people who held stigmatizing attitudes toward PLWA were significantly less likely to get routine testing (AOR = 0.52, CI = 0.32–0.84).

### Experiences with Testing

Among those tested, 54% were tested at VCT centers, 26% at public hospitals, and the rest were tested in outpatient clinics or private hospitals. Sixty-two percent of participants who had undergone testing reported that they last tested in 2004 (after the introduction of routine testing). Almost all respondents who had been tested reported that they made the decision on their own to get tested (93%); however, 68% of participants believed that they could not refuse the HIV test whether or not they had made the initial decision to test. Ninety-eight percent reported no ill treatment related to testing, and an equal proportion claimed that they did not regret getting tested. Most participants had obtained the results of their tests (94%) and reported that confidentiality had been strictly maintained at the testing centers (95%). Nearly all participants (99%) denied that their partner had hit or threatened them for being tested. Ninety-six percent reported receiving pre-testing counseling, 90% reported receiving post-testing counseling, and 92% reported that their experience with testing led them to encourage others to undergo testing. Individuals who tested by VCT reported pre-test counseling more frequently than those who tested by routine testing (97% versus 93%, *p* = 0.04) and less poor treatment from others related to testing (2% versus 6%, *p* = 0.03).

### Barriers and Facilitators to Testing

Table 5 presents reported impediments to HIV testing among respondents who had not been tested ( *n* = 658). Participants indicated whether any of the listed factors served as a barrier for them; they could agree with multiple possible responses. Almost half agreed that a key barrier to testing was that they were “afraid to know” if they were HIV-positive. Forty-three percent reported that they had no reason to believe that they were infected, and 33% did not test because testing positive would force them to stop some of their sexual practices. There were several significant gender differences in the cited barriers to testing. Women were significantly more likely than men to report lack of permission from their spouse or partner (10% versus 3%, *p* < 0.01). Men were more likely to cite frequent migration (25% versus 15%, *p* = 0.01), not wanting to change sexual practices (39% versus 27%, *p* < 0.01), and concerns about lack of social supports if they tested positive (20% versus 12%, *p* < 0.01).

**Table 5:**
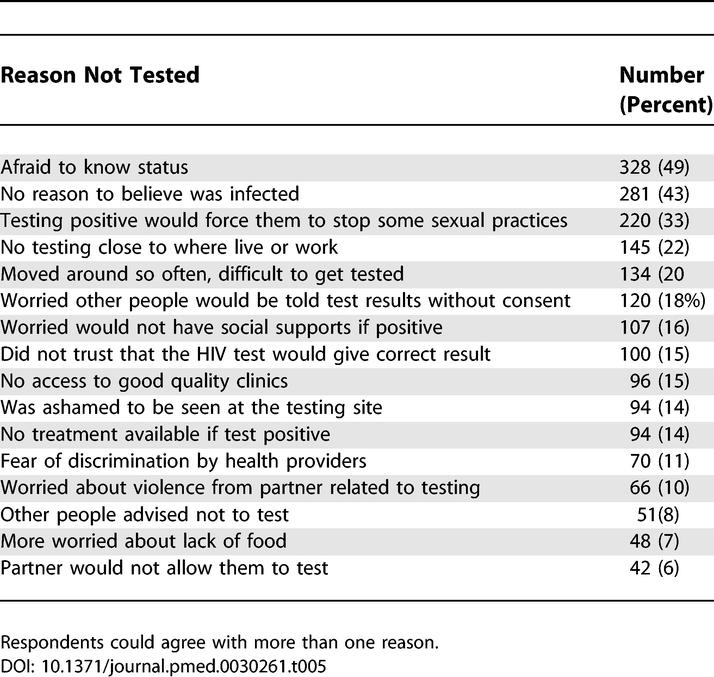
Principal Barriers to Testing among Respondents Who Had Not Been Tested ( *n* = 664)

Among those who had not been tested, 71% reported that they intended to be tested in the next six months. The most commonly cited factors that would facilitate testing included knowing that they could get treatment for HIV/AIDS (67%), and being tested with their spouse or main partner (64%). In a multivariate logistic model assessing planning to test as the dependent outcome and including all of our hypothesized correlates of getting tested ( Figure 1), respondents with stigmatizing attitudes had significantly lower odds of planning to get tested than those without stigmatizing attitudes (AOR = 0.44, 95% CI = 0.28–0.70). Respondents who reported unprotected sex had significantly higher odds of planning to test (AOR = 2.21, 95% CI = 1.42–3.44). The only other respondent characteristics associated with planning to test were urban location and self-reported very good or good health.

The most common facilitating factors among those tested were TV or radio messages (69%), knowing that treatment was available (65%), and knowing that the test results would be confidential (64%) ( Table 6). Men were significantly more likely than women to list treatment availability (74% versus 58%, *p* < 0.01), advice from family or friends (44% versus 34%, *p* = 0.02), messages from the media (77% versus 63%, *p* < 0.01), encouragement or support from someone who had been tested (55% versus 33%, *p* < 0.01), and confidentiality of testing (74% versus 56%, *p* < 0.01) as factors that influenced them to get tested. Women were significantly more likely to report encouragement from prenatal programs (31% versus 13%, *p* < 0.01) as a facilitator to testing.

**Table 6:**
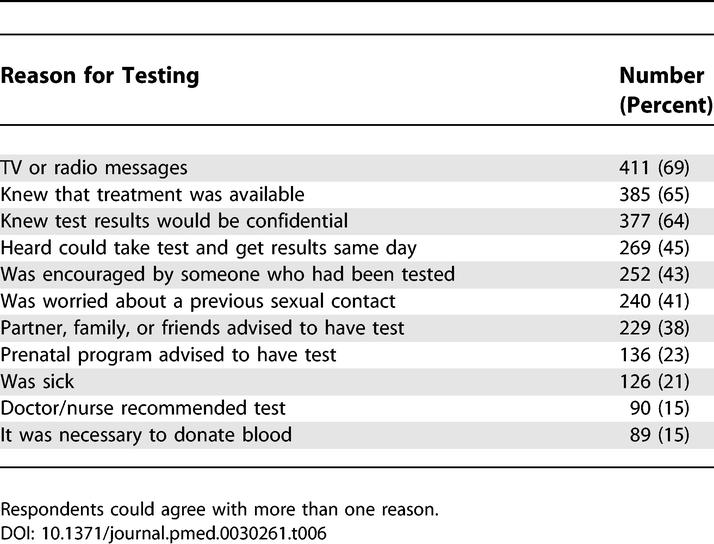
Principal Facilitators to Testing among Respondents Who Had Been Tested ( *n* = 590)

## Discussion

This is the first study to our knowledge to assess knowledge, attitudes, and experiences surrounding the new policy of routine testing in Botswana. We found that 11 months after the introduction of routine testing in Botswana, there was widespread support for this policy in a population-based survey, with 81% of participants reporting that they were either extremely or very much in favor of routine testing and an additional 8% reporting that they were somewhat in favor of this policy. A majority of respondents felt that routine testing would decrease barriers to testing, HIV-related stigma, and violence toward women, and would increase uptake of ARVs through the Botswana National Treatment Program. These results, in conjunction with data showing a significant increase in testing and treatment uptake since the introduction of this policy [ 9, 12, 44], suggest that this policy is beneficial in improving access to testing and life-saving treatment. Consistent with this, a study of several prenatal clinics in Botswana found that routine prenatal HIV testing was associated with a 15% increase in the proportion of pregnant women undergoing HIV testing between February and April 2004 (after routine testing was introduced) when compared with the last few months of 2003 [ 44]. Figures also indicate a more than 2-fold increase in treatment enrollment since the introduction of this policy, with 42,000 individuals enrolled in treatment as of March 2005 [ 45].

Evaluating our findings in the context of potential human rights burdens, we found that few individuals reported violence (1%), discrimination (2%), or a breach of confidentiality by healthcare workers (5%) associated with VCT or routine testing. Highlighting some potential problems with routine testing, 43% believed that routine testing would lead to avoidance of doctor visits for fear of being tested, and 14% felt that this policy could lead to increased violence against women. In addition, approximately two-thirds of participants who were tested by either routine testing or VCT felt that they could not refuse the HIV test, suggesting that the voluntary nature of both routine testing and VCT is not fully understood. These findings underscore the importance of implementing HIV testing policies with measures in place to ensure informed consent, protection of confidentiality, and protection of women from gender-based violence related to testing. Careful monitoring and evaluation of Botswana's routine testing program will help to ensure that the significant benefits of this program in terms of linkage to care and prevention of HIV transmission are not associated with potential adverse impacts.

Detailed guidelines for the implementation of routine testing were not introduced until February 2004, and the training of healthcare practitioners and the development of training materials were still ongoing in early 2005 [ 15]. Consequently, at the time of our study, there was still some confusion surrounding the details of implementation of this policy, including the extent to which routine testing should be provided as opt-out (all patients are automatically tested unless they refuse) or as routine-offer (all patients are offered a test, and they must provide explicit informed consent). The current policy has moved toward routine-offer HIV testing in accordance with the recommendations of WHO and UNAIDS; both organizations emphasize that the central principles of testing should include confidentiality, counseling, and informed consent [ 22, 24, 47]. As counseling has been found to account for some of the benefits of VCT in terms of reduced HIV transmission risk behavior [ 46] and linkage to subsequent medical care, reinforcing the importance of counseling in routine testing programs may help ensure that these programs help to maximize sexual-risk reduction and access to care. Additional conditions should be considered when implementing routine testing policies elsewhere, including the need to increase human resources and to expand the use of rapid testing.

Consistent with the documented role of HIV-related stigma as an impediment to testing in studies in Africa and elsewhere [ 6, 31, 37, 48– 50], we found that HIV-related stigma was associated with decreased odds of having been tested for HIV, of getting routine testing, and of planning to test among people not previously tested. In addition, respondents with more stigmatizing views about HIV and a greater number of fears related to HIV stigma were significantly less likely to have heard of routine testing after adjusting for possible confounders, attesting to the association between poor information and HIV-related stigma. Addressing HIV-related stigma should comprise an integral part of ongoing HIV testing programs and policies in Botswana, including measures to protect people with HIV/AIDS from discrimination in healthcare, work, and other settings. Policies that target HIV-related stigma may also prevent a reduction of clinical visits related to people's fears of being tested. Increasing testing and decreasing stigma will likely work together to reinforce one another, with more testing leading to a reduction in HIV-related stigma, which in turn will work to further increase testing. Botswana already has several innovative programs in place aimed to address stigma directly, including media campaigns, the public testing of President Festus Mogae and other national leaders, and the annual “Miss HIV Stigma Free” competition [ 11, 14]. Additional progress toward stigma reduction will require a deeper understanding of the structural dimensions of HIV-related stigma, and the mechanisms by which stigma reinforces and generates social inequalities related to gender, ethnicity, and class [ 51].

We found a relatively high prevalence of self-reported HIV testing in Botswana in the era of routine testing, compared with its neighboring countries. While 48% of our sample reported having been tested for HIV, results from Zimbabwe suggest that only 10%–12% of people are aware of their HIV status [ 52], and a nationwide community based–survey in South Africa in 2002 found that only 20% of people aware of VCT services had been tested for HIV [ 53]. In addition to the policy of routine testing, universal access to ARVs and to HIV testing likely contributes to the relatively high prevalence of testing in Botswana. Consistent with this, perceived access to testing was associated with 60% higher odds of having received an HIV test among respondents in our study, and the availability of ART was cited as a leading facilitator to testing. In addition, a national survey from Botswana in 2001 showed that fewer than 20% of individuals ages 15–49 had ever received an HIV test [ 54], suggesting a more than 2-fold increase in testing prevalence since the introduction of both universal ART access and routine testing. On the other hand, because over 50% of our sample had not yet been tested, our results reinforce the fact that availability of testing facilities and ART, while essential, may not be sufficient to guarantee HIV testing for many [ 31].

Study results should be interpreted in the context of a number of limitations. First, as this study was cross-sectional, causality cannot be determined from our findings. Second, while we interviewed individuals from both rural and urban areas, and covered the five most populated districts in Botswana, because we did not interview individuals in all districts of Botswana, our results may not be generalizable to the entire Botswana population. In addition, Botswana has a number of unique features that may limit generalizability to neighboring African countries, such as its relatively high per capita income, comparatively extensive healthcare infrastructure, strong donor involvement, and strong government commitment to combating HIV. Third, as the policy of routine testing was not yet implemented in a uniform way across all medical facilities in Botswana, and different facilities were at different stages of implementation, it was impossible to conduct a more systematic evaluation of the impacts of this policy. Moreover, since routine testing is a relatively new policy in Botswana, only a small proportion of those tested (15%) had been tested by routine testing at the time of our study. Finally, self-report can introduce misclassification and bias. To maximize validity we did not ask about HIV status, assured confidentiality and privacy, and asked survey questions in a culturally sensitive, nonjudgmental manner. To reduce social desirability bias, interviewers were not informed of key research hypotheses, and study aims were presented to participants in general terms.

### Concluding Remarks

In the face of a devastating epidemic that has already infected nearly half of its adult population, the government of Botswana has taken strong steps to improve access to testing and to ensure the right to life-sustaining treatment for all of its citizens. Early evidence of widespread support for the policy of routine testing in this study holds significant promise for the prevention and treatment of HIV/AIDS in Botswana and elsewhere. Concerted efforts to scale up HIV testing, however, must also be accompanied by appropriate monitoring of testing practices to ensure that they are implemented in accordance with international guidelines on human rights and HIV/AIDS [ 55, 56].

## Supporting Information

Protocol S1 Botswana Community Survey (338 KB DOC)

## Abbreviations

ARV: antiretroviral
ART: antiretroviral treatment
PLWA: people living with HIV and AIDS
VCT: voluntary counseling and testing
