# Correction: Routine HIV Testing in Botswana: A Population-Based Study on Attitudes, Practices, and Human Rights Concerns

**DOI:** 10.1371/journal.pmed.0030395

**Published:** 2006-10-31

**Authors:** Sheri D Weiser, Michele Heisler, Karen Leiter, Fiona Percy-de Korte, Sheila Tlou, Sonya DeMonner, Nthabiseng Phaladze, David R Bangsberg, Vincent Iacopino

In *PLoS Medicine*, volume 3, issue 7: DOI: 10.1371/journal.pmed.0030261

Table 3 should have listed Age (years) as Mean: 28.5, SD: 8.3 (not Mean: 8.3, SD: 9.6).

Minnehy Mukoma should have been included in the Acknowledgments for research design and implementation.

