# Supplementary material for: Routine HIV Testing in Botswana: A Population-Based Study on Attitudes, Practices, and Human Rights Concerns
Source: PLoS Med. 2006 Jul 18;3(7):e261. doi: 10.1371/journal.pmed.0030261 (PMC1502152; doi:10.1371/journal.pmed.0030261)
Supplement: Supplementary file 1 [file pmed.0030261.sd001.doc]

# Physicians for Human Rights and

# University of California, San Francisco

# Botswana Community Survey 2004

# #19 FINAL (11.25.04)

1. **Survey #**	(1-75) ID

2a. Date of interview	-	- 2004 DATE

2b. Interviewer code	(1, 2, 3,….) ICD

3a. District code	DC

3b Enumeration area code	EAC

3c. Locality code	LOC

4. Participation Outcome: [Circle ONE]

Survey Complete	1 OUTC

Not Available (2 visits)	2

Refusal	3a=Lack Time; 3b=Fear Reprisal; 3c=Opposed to Study; 3d=Other

Unable to Complete	4a=Interrupted; 4b=Emotional; 4c=Safety; 4d= Lack of Privacy; 4e=Other

**INTRODUCE YOURSELF**

*Was this household interviewed for BAIS II (Botswana AIDS Impact Survey)?* **IF YES, GO TO NEXT HOUSE.**

*I am working with a non-governmental organization called Physicians for Human Rights (PHR) and the University of Botswana. PHR is an organization that promotes the health and human rights of all people. We are conducting a health survey of as many women and men as possible and would like to speak to one member of this household. The person we select to interview must be between 18 and 49 years-old, speak Setswana or English, and be a resident of Botswana. With your permission, I would like to know how many people like that, live in this household, then using a statistics table I will randomly select one member of your household and interview them in private.*

**LIST HOUSEHOLD MEMBERS AND CHOOSE PARTICIPANT**

**READ CONSENT FORM**

5. Consent provided [Circle ONE]:	No	0 CNST

Yes	1

**PARTICIPANT CHARACTERISTICS**

6a*.* Sex [Circle ONE]	Male	0 GEN

Female	1

6b. *I will begin by asking you for some information about yourself.* *What month and year were you born?*	(month/year) AGEN

6c. *Are you currently married or living with a man/woman with whom you have a sexual relationship?* [Circle ONE]

Currently married, living with spouse	1 MRST

Currently married, living with other sexual partner	2

Currently married, not living with spouse or any other sexual partner	3

Not married, living with sexual partner	4

Not married, living with non-sexual partner	5

Not married, not living with sexual partner	6

NR	99

7. *How many children or other people depend on you for support?*	(number) NDEP

8. *What is the highest year you completed in school?*	Not finished primary	1 SCHL

Finished primary	2

Junior certificate	3

High school (form 5)	4

Post-secondary diploma	5

Tertiary	6

9*. What is your main occupation?* [Circle ONE]	Professional, non-health worker	1 OCUP

Professional, health worker	2

Clerical or office worker	3

Entrepreneur (public or private)	4

Military/Police/Prison	5

Security Guard	6

Mining	7

Farming for Employer	8

Housewife	9

Cleaning Staff (non-domestic)	10

Domestic Work	11

Construction	12

Street Vendor	13

Subsistence Farming for Self	14

Retired	15

Student	16

Unemployed	17

Other (Specify)	18

10. *What is your average monthly household income (total wages/income earned by all living with you)?* (Show List) HINC

<400 Pula	1

P400-P1,000	2

>P1,000-P5,000	3

>P5,000-P10,000	4

>P10,000-P20,000	5

>P20,000-P30,000	6

>P30,000	7

11. *In the* ***PAST 12 MONTHS****, have you had problems getting enough food to eat*? [Circle ONE]

No	0 FOOD

Yes	1

DK	88

NR	99

12a. *During a typical week, how many days a week do you consume alcohol?*	(#days/week) ALDA

12b. *On the days that you drink alcohol, how many drinks do you usually have?*	(#drinks/drinking day) ALDR

12c. *During a typical week, how often do you feel drunk?*	(#times/drunk/week) ALDK

13. *How would you describe your overall health?* [Circle ONE]

*Very Good/Excellent*	1 HSTAT

*Good*	2

*Fair*	3

*Poor*	4

DK	88

NR	99

##### KNOWLEDGE REGARDING HIV/AIDS

*14. I will now ask you some questions about what you know about HIV and AIDS. Based on what* ***you*** *know about AIDS, do* ***you*** *think that a person can get HIV or AIDS from any of the following? [READ ALL: Circle YES or NO for 14a-14j] TRANS*

| **Statement** | **Yes** | **No** | **DK** | **NR** |
| --- | --- | --- | --- | --- |
| a. *Having sexual intercourse without a condom (For the purpose of this survey, sexual intercourse is defined as vaginal or anal sex)* | 1 | 0 | 88 | 99 |
| b. *Using public toilets* | 1 | 0 | 88 | 99 |
| c. *Receiving a blood transfusion* | 1 | 0 | 88 | 99 |
| d. *Sharing used needles or instruments* | 1 | 0 | 88 | 99 |
| e. *Sharing meals with an HIV-positive person* | 1 | 0 | 88 | 99 |
| f. *During childbirth from mother to baby* | 1 | 0 | 88 | 99 |
| g. *During breastfeeding* *from mother to baby* | 1 | 0 | 88 | 99 |
| h. *Getting bitten by a mosquito* | 1 | 0 | 88 | 99 |
| i. *Shaking hands* | 1 | 0 | 88 | 99 |
| j. *Kissing* | 1 | 0 | 88 | 99 |

15. *Based on what you know of HIV and AIDS, which of the following do* ***you*** *think can help prevent someone from becoming infected with HIV/AIDS?* [READ ALL: Circle YES or NO for 15a-15e] PREV

| **Statement** | **Yes** | **No** | **DK** | **NR** |
| --- | --- | --- | --- | --- |
| a. *Using a condom correctly every time you have sex* | 1 | 0 | 88 | 99 |
| b. *Using traditional medicine* | 1 | 0 | 88 | 99 |
| c. *Being faithful with one uninfected partner* | 1 | 0 | 88 | 99 |
| d. *Praying* | 1 | 0 | 88 | 99 |
| e. *Not having sex at all* | 1 | 0 | 88 | 99 |

**TESTING AVAILABILITY**

16. *Do* ***you*** *have access to HIV testing (VCT or’Tebelopele’)?* [Circle ONE]

No	0 ACCT

Yes	1

DK	88

NR	99

17. *Is it possible in* ***your*** *village for someone to get a confidential test to find out if they are infected with HIV? By confidential, I mean that no one will know the test result, except you and your doctor, if you don’t want them to know?* [Circle ONE]

No	0 CNFT

Yes	1

DK	88

NR	99

##### TREATMENT AVAILABILITY

18. *Is* *treatment for AIDS, known as Antiretroviral (ARV) therapy, available in or near* ***your*** *village?* [Circle ONE]

No	0 AVAIL

Yes	1

DK	88

NR	99

19. *If you had HIV or AIDS, would ARVs be available to* ***you****?* [Circle ONE]

No	0 AVAILY

Yes	1

DK	88

NR	99

20. *Do you think there is less discrimination (bad treatment) of people with HIV/AIDS since ARVs became available in Botswana?* [Circle ONE]	No	0 STIGT

Yes	1

DK	88

NR	99

##### HIV TESTING

21. *I will now ask you some questions about HIV testing. I will* ***not*** *ask you to tell me your HIV status. If* ***you*** *were HIV positive, would you tell your sexual partner(s) your status*? [Circle ONE]

No	0 TELLP

Yes	1

DK	88

NR	99

22. *If* ***you*** *were to test positive for HIV and told others your status, do you think any of the following may happen to* ***you****?* [READ ALL: Circle YES or NO for 22a-22j] FEARS

| **Statement** | **Yes** | **No** | **DK** | **NR** |
| --- | --- | --- | --- | --- |
| a. *Break-up of your marriage or relationship* | 1 | 0 | 88 | 99 |
| b. *Physical abuse by your spouse/partner(s)* | 1 | 0 | 88 | 99 |
| c. *You would lose your job* | 1 | 0 | 88 | 99 |
| d. *You would be treated badly at work or school* | 1 | 0 | 88 | 99 |
| e. *Loss of your friends* | 1 | 0 | 88 | 99 |
| f. *Disowned from or neglected by your family* | 1 | 0 | 88 | 99 |
| g. *Treated badly by health professionals* | 1 | 0 | 88 | 99 |
| h. *Your community(village) would treat you like a social outcast* | 1 | 0 | 88 | 99 |
| i. *Your family would not care for you if you became sick* | 1 | 0 | 88 | 99 |
| j. *Other (please specify)* | 1 | 0 | 88 | 99 |

23. *If your spouse or one of your partner(s) was HIV-positive, how would* ***you*** *react to their status? Please let me know whether you agree or disagree with each of the following statements.* [READ ALL: Circle AGREE or DISAGREE for 23a-23h] REACT

| **Statement** | ***Agree*** | ***Disagree*** | **DK** | **NR** |
| --- | --- | --- | --- | --- |
| a. *You would get a test immediately* | 1 | 0 | 88 | 99 |
| b. *You would kick your spouse/partner out of the home* | 1 | 0 | 88 | 99 |
| c. *You would assume that your spouse/partner was having sex with other people* | 1 | 0 | 88 | 99 |
| d. *You would be grateful that he/she had told you* | 1 | 0 | 88 | 99 |
| e. *You may try to hit or hurt your spouse/partner* | 1 | 0 | 88 | 99 |
| f. *You would use condoms always if you continued to stay with him/her* | 1 | 0 | 88 | 99 |
| g. *You would not have sex anymore with your spouse/partner* | 1 | 0 | 88 | 99 |
| h. *Other (please specify)* | 1 | 0 | 88 | 99 |

**ROUTINE TESTING**

24a. *Have you ever heard of routine testing? Routine testing is a new approach to HIV testing announced in January 2004. It means that almost everyone who visits a health clinic or hospital will get a number of tests, including an HIV test, unless they say no to it.* [Circle ONE]	No	0 OPT

Yes	1

DK	88

NR	99

24b. *Now that you know what routine testing means, how much are* ***you*** *in favor of routine testing?* [Circle ONE]

*Extremely*	1 OPT1

*Very much*	2

*Somewhat*	3

*Not really*	4

*Not at all*	5

DK	88

NR	99

25. *We want to ask your opinion of routine testing. I will read you statements, let me know whether you agree or disagree.* [READ ALL: Circle YES or NO for 25a-25f] OPTR

| **Statement** | **Yes** | **No** | **DK** | **NR** |
| --- | --- | --- | --- | --- |
| a. *Routine testing results in less discrimination (bad treatment) of people who are HIV-positive* | 1 | 0 | 88 | 99 |
| b. *Routine testing makes it easier for people to get tested* | 1 | 0 | 88 | 99 |
| c*. Routine testing makes it harder for people to get tested* | 1 | 0 | 88 | 99 |
| d. *Routine testing leads to more violence against women* | 1 | 0 | 88 | 99 |
| e. *Routine testing leads to less violence against women* | 1 | 0 | 88 | 99 |
| f. *Routine testing helps people get access to ARV treatment* | 1 | 0 | 88 | 99 |
| g. *Routine testing will cause people to avoid seeing the doctor or nurse for fear of being tested* | 1 | 0 | 88 | 99 |

26. *Have you ever been tested for HIV*? [Circle ONE]	No	0 [GO TO 27] TEST

Yes	1 [GO TO 32]

DK	88[GO TO 27]

NR	99 [GO TO 27]

**FOR PEOPLE NOT TESTED**

27. *People have different beliefs about their risks for getting HIV.* *In your opinion, how would you rate your chances of becoming infected with HIV?* [Circle ONE]	*No risk*	1 RISK

*Low risk*	2

*Average risk*	3

*High risk*	4

*100% chance*	5

DK	88

NR	99

28. *Which of following prevented you from getting tested? I will read some statements, let me know if you agree or disagree.* [READ ALL: Circle YES or NO for 28a-28q] ROLE

| **Statement** | **Yes** | **No** | **DK** | **NR** |
| --- | --- | --- | --- | --- |
| a. *You were afraid to know if you are HIV- positive* | 1 | 0 | 88 | 99 |
| b. *You were ashamed to be seen at the testing site and believe that it will cause others to treat you badly (stigma)* | 1 | 0 | 88 | 99 |
| c. *Your spouse/partner(s) did not allow you* | 1 | 0 | 88 | 99 |
| d. *You were worried that your spouse/partner(s) would hit you or hurt you if he/she found out you were positive* | 1 | 0 | 88 | 99 |
| e. *You had no reason to believe that you were infected* | 1 | 0 | 88 | 99 |
| f. *You were worried that other people would be told your test results without your permission* | 1 | 0 | 88 | 99 |
| g. *There was no testing available close to where you live or work* | 1 | 0 | 88 | 99 |
| h. *You moved around so often, it was difficult to get tested* | 1 | 0 | 88 | 99 |
| i. *You knew that testing positive for HIV would force you to stop some of your sexual practices* | 1 | 0 | 88 | 99 |
| j. *Other people advised you not to get tested* | 1 | 0 | 88 | 99 |
| k. *Because health care providers would treat you badly if you were HIV-positive* | 1 | 0 | 88 | 99 |
| l. *You did not have access to good quality clinics* | 1 | 0 | 88 | 99 |
| m. *Because you did not trust that the HIV test would give the correct result* | 1 | 0 | 88 | 99 |
| n. *You thought that there is no treatment for HIV that would be available to you so there would be no point in having a test.* | 1 | 0 | 88 | 99 |
| o. *You were more worried about lack of food than about getting tested* | 1 | 0 | 88 | 99 |
| p. *You were worried that you would not have social supports* | 1 | 0 | 88 | 99 |
| q. *Other (please specify)* | 1 | 0 | 88 | 99 |

29. *What was the most important reason that made you not get tested?*	(# from 28a-28q; if OTHER, record response in English (specify)	) PREVI

30. *Do you intend to get tested for HIV within the* ***NEXT SIX MONTHS****?* [Circle ONE]

No	0 PLAN

Yes	1

DK	88

NR	99

31a. *Which of the following would help convince YOU to get tested?* [READ ALL: Circle YES or NO for 31a-31m] CONV

| **Statement** | **Yes** | **No** | **DK** | **NR** |
| --- | --- | --- | --- | --- |
| a. *If you were tested together with your spouse or main partner* | 1 | 0 | 88 | 99 |
| b. *If you had more trust in your doctor* | 1 | 0 | 88 | 99 |
| c. *If you knew that no one would find out your test results without your permission* | 1 | 0 | 88 | 99 |
| d. *If there was better counseling at the testing sites* | 1 | 0 | 88 | 99 |
| e. *If your spouse/partner would support your decision to get tested* | 1 | 0 | 88 | 99 |
| f. *If you knew that you could get treatment for HIV/AIDS (ARVs)* | 1 | 0 | 88 | 99 |
| g. *If health care providers would treat you well if you were HIV-positive* | 1 | 0 | 88 | 99 |
| h. *If there were better heath care facilities near you* | 1 | 0 | 88 | 99 |
| i. *If you could be tested and get the test results on the same day* | 1 | 0 | 88 | 99 |
| j. *If there were testing sites closer to your home or work* | 1 | 0 | 88 | 99 |
| k. *If there were a National HIV Testing week when leaders in society such as politicians, clergy, chiefs, celebrities and sports stars would get tested* | 1 | 0 | 88 | 99 |
| l. *If you had enough food to eat it would be easier for you to think about getting tested* | 1 | 0 | 88 | 99 |
| m. *Other (please specify)* | 1 | 0 | 88 | 99 |

31n. *What is the most important thing that would convince you to get tested?*	(# from 31a-31m; if OTHER, record response in English (specify)	) CONVM [NOW GO TO 36]

**FOR PEOPLE WHO HAVE BEEN TESTED**

32a. *Were you ever tested as part of routine testing*? [Circle ONE]

No	0 PLAN

Yes	1

DK	88

NR	99

**For people who were tested more than once, responses for 32b-35 refer to the MOST RECENT time they were tested.**

32b. *The following questions on HIV testing refer only to the* **MOST RECENT** *time you were tested. When were you last tested for HIV?*	(month/year) LASTT

32c. *Where were you tested?* [Circle ONE]	Public Hospital	1 WHERE

Private Hospital	2

TB Clinic	3

Pre-natal Clinic	4

Other Clinic	5

NGO	6

Private Laboratory	7

VCT Center	8

Other (specify)	9

DK	88

NR	99

32d *Did you make the decision to get the HIV test?* [Circle ONE]

No	0 WILLT

Yes	1

DK	88

NR	99

32e*.Did you feel you could say no to getting the HIV test?* [Circle ONE]

No	0 REFUS

Yes	1

DK	88

NR	99

32f. *Did others treat you badly because you got tested?* [Circle ONE]

No	0 DISCT

Yes	1

DK	88

NR	99

32g. *Do you regret getting tested?* [Circle ONE]	No	0 REGR

Yes	1

DK	88

NR	99

32h. *Did you find out the results of your test?* [Circle ONE]	No	0 REST

Yes	1

DK	88

NR	99

32i. *Did anybody find out your HIV test results from the testing center or your doctor without your permission?* [Circle ONE]	No	0 PERM1

Yes	1

DK	88

NR	99

32j. *Did your partner know you were tested?*	No	0 PKTS [GO TO 32l]

Yes	1 [GO TO 32k]

No partner	2 [GO TO 32l]

DK	88[GO TO 32l]

NR	99[GO TO 32l]

32k. *Did your partner hit you, kick you, hurt you in anyway, or threaten you because you were tested?* [Circle ONE]

No	0 TESTV

Yes	1

DK	88

NR	99

32l. *Did your experience with HIV testing lead you to encourage others to get tested?* [Circle ONE]

No	0 CONVT

Yes	1

DK	88

NR	99

**PRE TEST COUNSELING**

33a. *Did you receive counseling* ***before*** *being tested for HIV?* [Circle ONE]

No	0 COUNB

Yes	1

DK	88

NR	99

**POST-TEST COUNSELING**

33b. *Did you receive counseling* ***after*** *the HIV test*? [Circle ONE]

No	0 COUNA

Yes	1

Did not go back for results   	2

DK	88

NR	99

34. *What made* ***you*** *decide to have an HIV test?* (READ ALL: Circle YES or NO for 34a-34r) FACT

| **Statement** | **Yes** | **No** | **DK** | **NR** |
| --- | --- | --- | --- | --- |
| a. *You did not decide, the test was done without your consent* **[if YES, GO TO 36]** | 1 | 0 | 88 | 99 |
| b. *The doctor/nurse recommended it to you* | 1 | 0 | 88 | 99 |
| c. *You knew that treatment (ARVs) was available* | 1 | 0 | 88 | 99 |
| d. *You were sick* | 1 | 0 | 88 | 99 |
| e. *You knew that the test result would be confidential (that no one would find out the test result if you did not want them to know)* | 1 | 0 | 88 | 99 |
| f. *Your partner, family or friends advised you to have a test* | 1 | 0 | 88 | 99 |
| g. *TV or radio messages* | 1 | 0 | 88 | 99 |
| h. *People at church advised you to have a test* | 1 | 0 | 88 | 99 |
| i. *It was necessary for your job* | 1 | 0 | 88 | 99 |
| j. *It was necessary* *to donate blood* | 1 | 0 | 88 | 99 |
| k. *The prenatal (PMTCT) program advised you or your partner to have a test* | 1 | 0 | 88 | 99 |
| l. *It was required for military service* | 1 | 0 | 88 | 99 |
| m. *You were worried about a sexual contact* | 1 | 0 | 88 | 99 |
| n. *You heard that you can take the test and get the results in the same day* | 1 | 0 | 88 | 99 |
| o. *It was necessary to apply for health insurance or life insurance* | 1 | 0 | 88 | 99 |
| p. *You were encouraged and supported by someone who had been tested* | 1 | 0 | 88 | 99 |
| q. *Chiefs, religious leaders or political leaders got tested and this convinced you* | 1 | 0 | 88 | 99 |
| r. *Other (please specify)* | 1 | 0 | 88 | 99 |

35. *What was the most important reason that you got tested?*	(# from 34b-34r; if OTHER, record response in English (specify)	) FACTT

**STIGMA AND ATTITUDES TOWARDS PLWA**

36a. *I will now ask you some questions about people living with HIV or AIDS. Would you be willing to share a meal with a person you believed had HIV or AIDS?* [Circle ONE]

No	0 SHAR

Yes	1

DK	88

NR	99

36b. *If a relative of yours became ill with HIV/ AIDS, would you be willing to care for him or her in your household?* [Circle ONE]	No	0 RELM

Yes	1

DK	88

NR	99

36c. *If a student has HIV but is not sick, should he or she be allowed to continue attending school?* [Circle ONE]

No	0 SCHLS

Yes	1

DK	88

NR	99

36d. *If a teacher has HIV but is not sick, should he or she be allowed to continue teaching in school?* [Circle ONE]

No	0 SCHLT

Yes	1

DK	88

NR	99

36e. *If you believed a shopkeeper or food seller had the AIDS virus, would you buy food from him or her?* [Circle ONE]

No	0 SHOP

Yes	1

DK	88

NR	99

36f. *If a member of your family became ill with HIV/AIDS, would you want it to remain secret?* [Circle ONE]	No	0 FSEC

Yes	1

DK	88

NR	99

37. *Do you think that people with HIV/AIDS should have the same rights as people who do not have the disease?* [Circle ONE]	No	0 RGHT

Yes	1

DK	88

NR	99

**HEALTH CARE EXPERIENCES**

38a. *I will now ask you about your use of health care services. Where do you get most of your health care?* [Circle ONE]

Public clinic	1 HEALT

Private clinic	2

Hospital	3

Traditional healer	4

Other (specify):	5

38b. *How often in the* ***PAST 12 MONTHS*** *did you see a medical doctor?* [Circle ONE]

Never	1 OFTEN

1-2 times	2

3-4 times	3

More than 4 times	4

38c. *Do you have access to good quality medical* *services?* [Circle ONE]

No	0 ACCS

Yes	1

DK	88

NR	99

39. *Have you experienced any of the following from a doctor or nurse over the* ***PAST 12 MONTHS****?* [READ ALL: Circle YES or NO for 39a-39e] HPABS

| **Statement** | **Yes** | **No** | **DK** | **NR** |
| --- | --- | --- | --- | --- |
| a. *Denial* *of* *care or treatment* | 1 | 0 | 88 | 99 |
| b. *Denial of admission to a hospital* | 1 | 0 | 88 | 99 |
| c. *Verbal mistreatment or insults* | 1 | 0 | 88 | 99 |
| d. *Usually* *treated with respect and dignity* | 1 | 0 | 88 | 99 |
| e. *Other mistreatment* *(specify)* | 1 | 0 | 88 | 99 |

##### SEXUAL PRACTICES

*As you may know, a person may get the AIDS virus through sexual activity. To help prevent the spread of HIV/AIDS, we need to know more about the different types of sexual practices people engage in. Some of these questions need to be rather detailed and personal. Since this survey is confidential, no one will know your answers. I would appreciate your cooperation and honesty* *in answering these questions.*

40. *Have you* ***EVER*** *had sexual intercourse? For the purpose of this survey, sexual intercourse is defined as vaginal or anal sex with either a man or a woman.* [Circle ONE]	No	0 [GO TO 44, THEN TO 48] EVER

Yes	1 [FOR ALL OTHER, GO TO 41]

DK	88

NR	99

41. *In your lifetime, have you ever been forced to have sex when you did not want to?* [Circle ONE]

No	0 FFSX

Yes	1

DK	88

NR	99

42. *The number of sexual partners people have had differs a lot from person to person. Some people report having had one sexual partner, some two partners, and others many* *partners. How many sexual partners have you had in the* ***PAST 12 MONTHS****?* [Circle ONE]	None	1 [GO TO 44, THEN TO 48] NUMB

One	2 [FOR ALL OTHER, GO TO 43]

Two	3

Three	4

Other (specify)	5

43. *How many sexual partners have you had in the* ***LAST MONTH****?* [Circle ONE]

None	1 [GO TO 44] NUMBLM

One	2 [FOR ALL OTHER, GO TO 45a]

Two	3

Three	4

Other (specify)	5

44. *Do you practice abstinence as a way to prevent yourself or others from becoming infected with HIV/AIDS?* [Circle ONE]

No	0 ABST

Yes	1

DK	88

NR	99

**FOR SEXUALLY ACTIVE PARTICIPANTS ONLY**

45a. *Who generally decides when you have sex?* [READ ALL: Circle ONE]	*You*	1 DCES

*You* *and your partner(s) equally*	2

*Your partner(s)*	3

DK	88

NR	99

45b. *Thinking about all the times you had sex in the* ***PAST 12 MONTHS****, how often did you drink alcohol before having sex?* [READ ALL: Circle ONE]	*Very infrequently or never*	1 ALCS

*Less than half of the time*	2

*More than half of the time*	3

*Very frequently or always*	4

DK	88

NR	99

45c. *Have you had sex with a commercial sex worker during the* ***PAST 12 MONTHS****?* [Circle ONE] COMSX

No	0

Yes	1

DK	88

NR	99

45d. *During the* ***PAST 12 MONTHS****, have you given another person money, food, or other resources in exchange for sex?*

[Circle ONE]	No	0 PAID

Yes	1

DK	88

NR	99

45e. *During the* ***PAST 12 MONTHS****, has another person given you money, food, or other resources in exchange for sex?* [Circle ONE]	No	0 EXCH

Yes	1

DK	88

NR	99

45f. *Were you forced to have sex against your will over the* ***PAST 12 MONTHS****?* [Circle ONE]	No	0

Yes	1

DK	88

NR	99

45g. *Did you have sex with others when they did not want to over the* ***PAST 12 MONTHS****?* [Circle ONE] FOSEX	No	0

Yes	1

DK	88

NR	99

45h. *To the best of your knowledge, have your sexual partners had sex with anyone else besides you over the* ***PAST 12 MONTHS****?* [Circle ONE]	No	0 PMONO

Yes	1

DK	88

NR	99

45i. *In* *the* ***PAST 12 MONTHS,*** *have you had a sexual relationship with someone 10 or more years older than you?* [Circle ONE]

No	0 INTGN

Yes	1

DK	88

NR	99

45j. *In the* ***PAST 12 MONTHS*** *have you had a sexual relationship with someone 10 or more years younger than you?* [Circle ONE]	No	0 INTGN1

Yes	1

DK	88

NR	99

45k. *We need to ask you about the sex of your sexual partner because some sexual practices can contribute to the spread of HIV. The sex of your sexual partners over* *the* ***PAST 12 MONTHS*** *includes:* [READ ALL: Circle ONE]	*Men Only*	1 SEXP

*Women Only*	2

*Usually Men*	3

*Usually Women*	4

*Both men and women*	5

DK	88

NR	99

REGARDING CONDOM USE

46a. *Now I will ask you about condom use. Have you and your spouse/primary partner discussed condom use? (For the purpose of this survey, primary partner means your partner in an exclusive sexual relationship or the person who is most often your sexual partner.)* [Circle ONE]	No	0 CONDD

Yes	1

No Partner	2

DK	88

NR	99

46b. *Thinking about all of the times you had sex over the* ***PAST 12 MONTHS****, how often did you use a condom?* [Circle ONE]	*All sexual encounters*	1 CONDT

*Most sexual encounters*	2

*Half of sexual encounters*	3

*A few sexual encounters*	4

*Never*	5 [GO TO 46d]

DK	88

NR	99

46c. *In your sexual encounters, who typically decides whether you use a condom?* [Circle ONE]

*You only*	1 DECI

*Mostly you*	2

*You and your partner(s) equally*	3

*Mostly partner(s)*	4

*Partner(s) only*	5

Other (please specify)	6

46d. *Have you or your sexual partner(s) used the female condom?* [Circle ONE]	No	0 FCND

Yes	1

DK	88

NR	99

46e. *How many times did you have sex* ***without*** *a condom over the* ***PAST MONTH*** *with someone who is* ***not*** *your spouse or primary partner?*	(WRITE IN number of times) UNPRO

46f. *Was there anytime that you did* ***not*** *use a condom over the* ***PAST 12 MONTHS****?* [Circle ONE]

No	0 [GO TO 48] NCOND

Yes	1 [GO TO 47]

DK	88 [GO TO 48]

NR	99 [GO TO 48]

47. *Why do you* ***not*** *use a condom with each sexual encounter?* [READ ALL: Circle YES or NO for 47a-47k] CONDW

| **Statement** | **Yes** | **No** | **DK** | **NR** |
| --- | --- | --- | --- | --- |
| a. *Condoms are inconvenient to use* | 1 | 0 | 88 | 99 |
| b. *It decreases sexual pleasure* | 1 | 0 | 88 | 99 |
| c. *Your spouse/partner(s) does not want to* | 1 | 0 | 88 | 99 |
| d. *You have no control over whether your spouse/ partner(s) uses a condom* | 1 | 0 | 88 | 99 |
| e. *You or your spouse/partner(s) are trying to get pregnant* | 1 | 0 | 88 | 99 |
| f. *You cannot afford condoms* | 1 | 0 | 88 | 99 |
| g. *Condoms are not available in your area* | 1 | 0 | 88 | 99 |
| h. *You do not know how to use a condom* | 1 | 0 | 88 | 99 |
| i. *Condoms do not prevent HIV/AIDS* | 1 | 0 | 88 | 99 |
| j. *You use other birth control methods* | 1 | 0 | 88 | 99 |
| k. *Other (please specify)* | 1 | 0 | 88 | 99 |

**FOR ALL PARTICIPANTS**

48a.*If your partner wants to use a condom, do you think this means that they are having sex with other people?* [Circle ONE]

No	0 CONDS

Yes	1

DK	88

NR	99

48b. *If your partner wants to use a condom, do you think this means that they don’t trust you?* [Circle ONE]

No	0 CONDT

Yes	1

DK	88

NR	99

##### GENDER ROLES

49. *I will now read you some statements about your beliefs about the different roles of men and women in society. Please let me know whether you agree or disagree with each of the following.* [READ ALL: Circle AGREE or DISAGREE for 49a-49o] GEND

| **Statement** | ***Agree*** | ***Disagree*** | **DK** | **NR** |
| --- | --- | --- | --- | --- |
| a. *It is OK for men to have more than one partner at one time* | 1 | 0 | 88 | 99 |
| b. *It is OK for women to have more than one partner at one time* | 1 | 0 | 88 | 99 |
| c. *A women must prove her fertility before she can marry* | 1 | 0 | 88 | 99 |
| d. *A woman should remain a virgin until she marries* | 1 | 0 | 88 | 99 |
| *e. A man should remain a virgin until he marries* | 1 | 0 | 88 | 99 |
| f. *A man must prove his fertility before he can marry* | 1 | 0 | 88 | 99 |
| g. *It is a wife’s duty to have sex with her husband even if she does not want to* | 1 | 0 | 88 | 99 |
| h. *It is more important for a woman to respect her spouse/partner than it is for a man to respect his spouse/partner* | 1 | 0 | 88 | 99 |
| i. *A man may beat his spouse/partner if she disobeys him* | 1 | 0 | 88 | 99 |
| j. *A man may beat his spouse/partner if he believes she is having sex with other men* | 1 | 0 | 88 | 99 |
| k. Women should be legally entitled to inherit their husband’s property/estate | 1 | 0 | 88 | 99 |
| l. Women should have the same legal rights as men | l | 0 | 88 | 99 |
| m. You support the practice of men paying a bride price in order to marry a woman | 1 | 0 | 88 | 99 |
| n. It is more important that boys get an education than that girls do | 1 | 0 | 88 | 99 |
| o. It is more important for women to get HIV tested than for men to get tested | 1 | 0 | 88 | 99 |

50. *Women get HIV more often than men do in Botswana. Why do you think this is? I will read you a number of reasons and please let me know if you agree or disagree with each one.* [READ ALL: Circle YES or NO for 50a-50o] VULNW

| **Statement** | **Agree** | **Disagree** | **DK** | **NR** |
| --- | --- | --- | --- | --- |
| a. *Women cannot say no to sex* | 1 | 0 | 88 | 99 |
| b. *Men refuse to use a condom* | 1 | 0 | 88 | 99 |
| c. *Women do not have enough power within relationships with their partner* | 1 | 0 | 88 | 99 |
| d. *Women cannot insist on using condoms if their partner does not want to* | 1 | 0 | 88 | 99 |
| e. *Domestic violence/rape* | 1 | 0 | 88 | 99 |
| f. *Relationships between young women and older men are common* | 1 | 0 | 88 | 99 |
| g. *Women have multiple sexual partners* | 1 | 0 | 88 | 99 |
| h. *Men have multiple partners and put women at risk* | 1 | 0 | 88 | 99 |
| i. *Women exchange sex for food, money or other resources* | 1 | 0 | 88 | 99 |
| j. *Women do not have equal rights to men* | 1 | 0 | 88 | 99 |
| k. *Women are valued less than men in society* | 1 | 0 | 88 | 99 |
| l. *Women are dependent upon men economically* | 1 | 0 | 88 | 99 |
| m. *Men drink too much alcohol which makes them act irresponsibly* | 1 | 0 | 88 | 99 |
| n*. Women drink too much alcohol which makes them act irresponsibly* | 1 | 0 | 88 | 99 |
| o. *Other (specify)* | 1 | 0 | 88 | 99 |

51. *What is the most important reason that women are at high risk for HIV?*	( # from 50a-50o; if OTHER, record response in English (specify)	) VULWO

52. *What is the most important reason that men are at high risk for HIV?*	(# from 50a-50o; if OTHER, record response in English (specify)	) VULMN

**HIV/AIDS IN BOTSWANA**

*53. Now I will ask you about what more can be done about HIV/AIDS in Botswana. In your opinion, what could be done to help more people get* ***treatment*** *(ARVs) for HIV/AIDS in Botswana?* [DO NOT READ: CIRCLE ALL THAT APPLY] MRTX	Improving education about treatment	1

Encouraging more people to get tested	2

Increasing confidentiality at treatment centers	3

Decreasing time delays at the treatment centers	4

Providing assistance with food	5

If doctors/nurses would treat people with HIV better	6

If people had access to better clinics and hospitals	7

If there were more HIV/AIDS support groups	8

Other (specify)	9

54. *Have political leaders done enough to address the problem of HIV/AIDS in Botswana?* [Circle ONE]

No	0 LEADP

Yes	1

DK	88

NR	99

55. *Has your village chief done enough to address the problem of HIV/AIDS?* [Circle ONE]

No	0 LEADC

Yes	1

NA (no chief)	2

DK	88

NR	99

56. *Have your church leaders done enough to address the problem of HIV/AIDS?* [Circle ONE]

No	0 LEADR

Yes	1

NA (no church leaders)	2

DK	88

NR	99

**ASSESSMENT FOR MAJOR DEPRESSION: Hopkin’s Symptom Check-List for Depression**

57. *The following* *are symptoms or problems that people sometimes have. Please let us know the extent to which each of the following symptoms has bothered you* *or distressed you in the* ***LAST WEEK****, including today.* [READ ALL: Select ONE ANSWER from options 1 to 4 for questions 57a-57o] DEPR

| **Statement** | ***Not at all*** | ***A little*** | ***Quite a bit*** | ***Extremely*** | **NR** |
| --- | --- | --- | --- | --- | --- |
| a. *Feeling low in energy, slowed down* | 1 | 2 | 3 | 4 | 99 |
| b. *Blaming yourself for things* | 1 | 2 | 3 | 4 | 99 |
| c. *Crying easily* | 1 | 2 | 3 | 4 | 99 |
| d. *Loss of sexual interest or pleasure* | 1 | 2 | 3 | 4 | 99 |
| e. *Poor appetite* | 1 | 2 | 3 | 4 | 99 |
| f. *Difficulty falling asleep, staying asleep* | 1 | 2 | 3 | 4 | 99 |
| g. *Feeling hopeless about the future* | 1 | 2 | 3 | 4 | 99 |
| h. *Feeling down or blue* | 1 | 2 | 3 | 4 | 99 |
| i. *Feeling lonely* | 1 | 2 | 3 | 4 | 99 |
| j. *Thoughts of ending your life* | 1 | 2 | 3 | 4 | 99 |
| k. *Feelings of being trapped or caught* | 1 | 2 | 3 | 4 | 99 |
| l. *Worry too much about things* | 1 | 2 | 3 | 4 | 99 |
| m*. Feeling no interest in things* | 1 | 2 | 3 | 4 | 99 |
| n. *Feeling everything is an effort* | 1 | 2 | 3 | 4 | 99 |
| o. *Feeling worthless* | 1 | 2 | 3 | 4 | 99 |

##### IF you circled 2-4 for 57j, GO TO 58; if not, GO TO 60.

58. *Based on your response, I am concerned about your safety. I would like you to speak with one of the doctors or nurses working on this study because they may be able to help you. While you are free to refuse, I want you to know that the doctor or nurse will not tell anyone else anything that you tell them without your permission. This health worker would like to help make sure that you are safe and that you know where to go to get help. Would you like to speak with a health worker?*	No	0 [GO TO 59] SUICP

Yes	1 [GO TO 60]

DK	88 [GO TO 59]

NR	99 [GO TO 59]

59. *I would like to provide you with some information on nearby hospitals or clinics that can help people who have thoughts about hurting themselves. Can I give you some information on where you can get help?*

No	0 SUICI

Yes	1

DK	88

NR	99

60. *If you would like any information on the availability of HIV testing in your community please let me know. Would you like some information?*	No	0 INFO

Yes, information provided	1

61. *If you would like information on how to prevent HIV transmission or on treatment for AIDS, I have a pamphlet with some information for you. Would you like a pamphlet?*	No	0

Yes, information provided	1 INFOP

FOR ALL PARTICIPANTS

62*. In Botswana, people sometimes don’t feel safe at home because they experience domestic violence. Domestic violence means that their partners hurt them (hit or kick them), threaten them or force them to have sex when they don’t want to. As part of our study, we would like to let you know who to contact if you or someone that you know does not feel safe at home. These organizations are here to protect people who are being hurt. We also want you to know that in Botswana every citizen has the right to report domestic violence to the police. Would you like information on how you can contact these organizations?*	No	0 FORCI

Yes, information provided	1
